# Prevalence of *Salmonella* in raw animal products in Ethiopia: a meta-analysis

**DOI:** 10.1186/s13104-015-1127-7

**Published:** 2015-04-21

**Authors:** Getachew Tadesse, Endrias Zewdu Gebremedhin

**Affiliations:** Department of Biomedical Sciences, College of Veterinary Medicine and Agriculture, Addis Ababa University, P.O. Box 34, Debre Zeit, Ethiopia; Department of Veterinary Laboratory Technology, Faculty of Agriculture and Veterinary Science, Ambo University, P.O. Box 19, Ambo, Ethiopia

**Keywords:** Animal products, Ethiopia, Markets, *Salmonella*, Slaughter houses

## Abstract

**Background:**

The contributions of animal products to human salmonellosis differ across countries, and source attribution is a major step in prioritizing control measures. The objectives of this study were to estimate the prevalence of *Salmonella* in raw animal products in Ethiopia by using meta-analytical methods.

**Results:**

The odds of *Salmonella* contaminated meat was more than twice higher in markets than in slaughter houses [Odds ratio (OR) = 2.25 (95% Confidence Interval [CI] = 1.75, 2.89)]. The source species significantly affected meat contamination in slaughter houses (*P* < 0.05) but not in the markets (*P* > 0.05). The pooled estimates of *Salmonella* contaminated goat carcasses, beef carcasses, minced beef and milk were 3.86%, 4.53%, 8.34% and 10.76% respectively.

**Conclusions:**

The estimates demonstrate the extent of contamination, and imply the need for safety intervention measures to reduce the risks of contamination of animal products and human illnesses.

**Electronic supplementary material:**

The online version of this article (doi:10.1186/s13104-015-1127-7) contains supplementary material, which is available to authorized users.

## Background

*Salmonella* is one of the major public health concerns all over the world. Whilst typhoidal infections are common in the tropics and subtropics where the sewage disposal system and the food handling hygienic standards are inadequate [1], non-typhoidal *Salmonella* (NTS) is important in both developed and developing countries. NTS enters the food chain at any point in livestock feed, and in food manufacturing, processing, retailing, catering and preparation [2]; survives typical catering refrigeration temperatures and increases in number under conditions of thermal abuse [3]. Apart from sporadic infections, outbreaks associated with the consumption of contaminated animal products have been recorded in several countries [4-9].

As the relative contributions of animal products to human salmonellosis differ across countries [10], source attribution is a major step in prioritizing control measures [11]. Elsewhere, different methods that included microbial sub-typing, comparative exposures, epidemiological analyses of sporadic cases and outbreaks, and expert elicitations have been used to attribute sources to human cases [10]. In Ethiopia, the incidence of food-borne salmonellosis is unknown; the risk factors associated with the contamination of animal products are not described, and there have not been studies on attribution of sources to human illnesses. However, the considerable occurrence of carrier food animals (7.07% in cattle to 43.81% in pigs) [12] and the wide spread raw animal product consumption habit in a noteworthy segment of the population are suggestive of the risk of acquiring *Salmonella* from animal products. Therefore, quantitative syntheses of studies’ estimates could enable to appreciate the level of contamination and the comparative importance of animal products as potential sources of *Salmonella* infections to humans. The objective of this study was to estimate the prevalence of *Salmonella* in raw animal products of Ethiopia by using meta-analytical methods.

## Methods

The study was conducted according to the guideline of the PRISMA (Preferred Reporting Items for Systematic Reviews and Meta-Analyses) group [13]. The PRISMA check list was used to ensure inclusion of relevant information (see Additional file 1).

### Literature search and study selection

The literature search strategy was described in a previous report [14]. Briefly, published studies were searched in Medline, and non-Medline indexed articles were searched in Google scholar and in the lists of references of articles. The last search was done on December 10, 2014. A study was screened for eligibility if (i) it was published in English, (ii) the samples were raw and (iii) collected from slaughter houses, ‘super markets’ or farms. A study was excluded if (i) the titles and abstracts were not relevant to the outcomes of interest, (ii) it was a duplicate and (iii) the methodology was not appropriate.

### Data extraction

From each eligible study, the first author, year of publication, year of study, location, study design, sample source, sample type, sample size, microbiological methods and numbers of *Salmonella* positive samples were extracted. The study level estimates and standard errors were derived from the extracted data.

### Data analysis

To produce conservative estimates, a zero reported for the number of positive samples was imputed as 0.5 [15]. The study level estimates were transformed to logit event estimates [16,17] by the following formula: lp = ln [p/ (1 − p)], where lp = logit event estimate; ln = natural logarithm; p = study level estimate. The variances of the estimates were calculated by the following formula: v (lp) = 1/ (np) + 1/ [n (1 − p)], where v = variance, and n = sample size.

### Assessment of bias

The qualities of the methods (sampling and microbiological) were used to assess the within study biases. The across study bias (small study effects) of the estimates on meat items was visually examined by a funnel plot, and the Egger’s regression asymmetry test was used to test the statistical significance of the bias [18]. The Duval and Tweedie non-parametric ‘fill and trim’ linear random method was used to calculate unbiased estimates [19].

### Heterogeneity analysis

The Galbraith plot was used to get a visual impression of the heterogeneity of the estimates on the prevalence of contaminated meats [20]. The significance of the heterogeneities was assessed by the Cochran’s Q test, and a non significant heterogeneity was accepted if the ratio of Q and the degree of freedom (Q/df) was less than one. The inverse variance index (I^2^) was used to estimate the percentage of the variation attributable to heterogeneity, and I^2^ values of 25%, 50% and 75% were considered as low, moderate and high heterogeneity, respectively [21]. Subgroup analyses were done by sample source (slaughterhouse/market), type of sample (whole muscle/swab) and source species.

### Pooled estimates

The DerSimonian and Laird random effects model was used to pool logit event estimates [22]. The pooled estimates were back transformed to prevalence estimates (p): p = e^lp^/ (e^lp^ + 1): where e = the base of natural logarithm. Single study omitted influence analyses were done to test the sensitivities of pooled estimates. A study was considered to be influential if the pooled estimate without it was not within the 95% confidence limits of the overall mean. The Z test was used to test whether a pooled estimate significantly differs from zero or not. The Chi Square test was used to test the significance of differences in pooled estimates [23,24]. Alpha was set at 0.05.

Microsoft Office Excel 2007 was used to calculate study level prevalence estimates, logit event estimates, standard errors and to back transform logit event estimates to proportions. Epi info™ (Version 3.5.1, Center for Disease Control, CDC, USA) was used to compare groups. Stata (Version 11.1, Stata Corp, College Station, Texas) was used in all other analyses.

## Results and discussion

### Search and selection of studies

**Figure 1:**
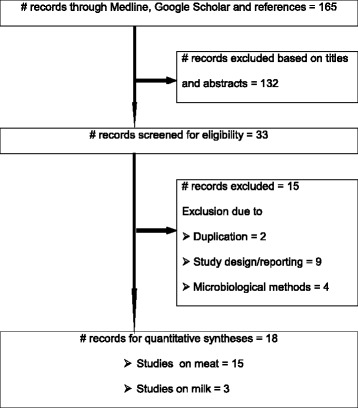
A flow diagram of the selection of eligible studies.

Figure 1 presents the search results. A total of 165 studies were found, and 128 studies were excluded on the basis of the titles and abstracts. Of the articles screened for eligibility, 15 were excluded due to diverse reasons: two studies were duplicates; one study was serotype specific; the samples were not raw in three studies; in one study, the samples were not neither from abattoirs nor ‘super markets’ nor farms; the sample sizes were small in two studies; in one study most samples were from a single farm; the exact number of positive samples was not reported in one study, and in four studies the microbiological methods were not appropriate. A total of 18 studies were eligible for quantitative syntheses [25-42].

### Characteristics of the eligible studies

Table 1 presents the characteristics of the eligible studies. The studies were conducted between 1999 and 2011 in Central, Eastern, Northern and Southern Ethiopia. Fifteen studies were on meat samples collected from abattoirs and/or ‘supermarkets’. Three studies were on milk samples collected from farms. Data from 3706 meat (beef, pork and mutton, goat, camel and chicken meats) and 395 cow milk samples were considered for quantitative syntheses. The study level estimates ranged from zero in goat meat to 21.01% in camel meat.

**Table 1:** **Characteristics of the eligible studies on** ***Salmonella*** **contaminated products**

**Author**	**Ys**	**Location**	**Sample**	**Source**	**n**	**Positive (%)**
[25]	1999	AA	Beef	Abattoir	235	23 (9.79)
[26]	1999-2000	DZ	Beef	Abattoir	323	9 (2.79)
[27]^a^	2005-2006	DZ	Beef	Abattoir	100	2 (2)
[28]^a^	2006-2007	BD	Beef	Abattoir	186	9 (4.84)
[29]	2002-2003	DZ	Goat meat	Abattoir	60	2 (3.33)
[30]	2003-2004	AM	Goat meat	Abattoir	100	0 (0)
[31]^a^	2007-2008	MD	Goat meat	Abattoir	60	5 (8.33)
[29]	2002-2003	DZ	Mutton	Abattoir	47	5 (10.64)
[30]	2003-2004	AM	Mutton	Abattoir	104	2 (1.92)
[31]	2007-2008	MD	Mutton	Abattoir	142	20 (14.08)
[32]^a^	2004-2005	AA	Pork	Abattoir	277	11(3.97)
[33]^b^	2004-2005	AA	Pork	Abattoir	99	2 (2.02)
[34]	2001-2002	DJ	Camel meat	Abattoir	119	25 (21.01)
[35]	2001	AA	Chicken meat	Market	244	30 (12.30)
[36]^c^	2001-2002	DA	Chicken meat	Market	104	16 (15.38)
[37]	2003-2004	AA	Chicken meat	Market	208	29 (13.94)
[25]	1999	AA	Minced beef	Market	330	26 (7.88)
[38]	2002-2003	AA	Minced beef	Market	160	23 (14.38)
[37]	2003-2004	AA	Minced beef	Market	142	12 (8.45)
[39]	2009	JM	Minced beef	Market	120	1 (0.83)
[38]	2002-2003	AA	Mutton	Market	85	12 (14.12)
[37]	2003-2004	AA	Mutton	Market	212	23 (10.85)
[38]	2002-2003	AA	Pork	Market	55	9 (16.36)
[37]	2003-2004	AA	Pork	Market	194	22 (11.34)
[40]	2010	AA	Milk	Farm	195	6 (3.08)
[41]	2010-2011	KR	Milk	Farm	100	20 (20)
[42]	nr	SB	Milk	Farm	100	16 (16)

AA, Addis Ababa; BD, Bahirdar; AM, Addis Ababa and Modjo; DJ, Diredawa and JiJiga; DZ, DebreZeit; JM, Jimma; KR, Kersa; MD, Modjo; n, Sample size; nr, not reported; Ys, year of study; SB, Sebetta; Ys, year of study.

^a^The samples were carcass swabs.

^b^The samples were mixed abdominal and diaphragmatic muscle samples.

^c^The samples were taken from a slaughtering plant and markets.

### Bias and heterogeneity

**Figure 2:**
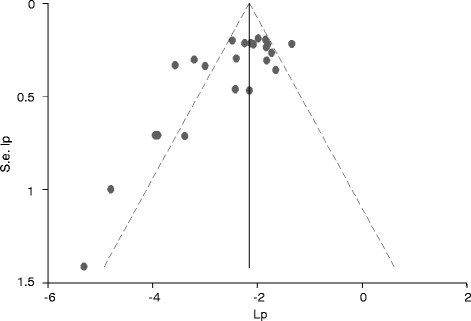
Funnel plot of the logit event estimates (lp) of *Salmonella* in meat items.

Sampling was random in thirteen studies [25,27-31,33,36,37,39-42]. In three studies, samples were taken from all animals presented for slaughter in each sampling day [26,32,34]. The sampling methods were not reported in two studies [35,38]. The analytical units were 25 g muscle samples [25,26,29,30,33-39], carcass swabs [27,28,31,32] and one ml milk [40], but not reported in two studies [41,42]. In all studies, *Salmonella* was isolated and identified as per the guideline of the International Organization for Standardization (ISO 6579, 1998-2002) with modifications. Serotypes were reported in 13 studies [25-30,32-38]. The funnel plot of the estimates of contaminated meat items was asymmetric (Figure 2) and the intercept of the regression of the standardized effect estimates against the precision significantly deviates from zero [bias coefficient = -3.23 (95% CI = -4.92, -1.53); *P* < 0.001]. The asymmetry of the plot was not due to small study effects, and theoretical missing studies were not incorporated by the Duval and Tweedie method. The plot and tests did not suggest the presence of bias.

**Figure 3:**
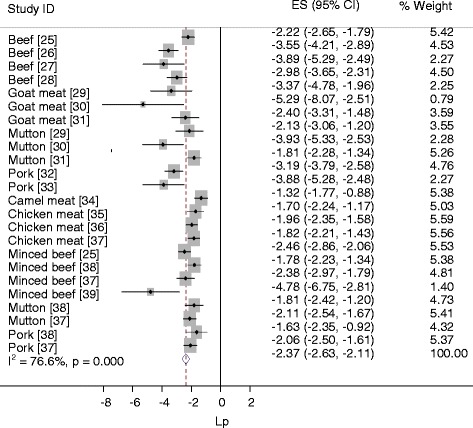
Forest plot of the logit event estimates (lp) of *Salmonella* in meat items.

**Figure 4:**
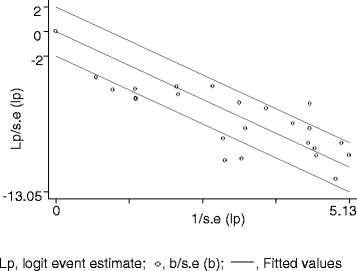
Galbraith plot of the logit event estimates (lp) of *Salmonella* in meat items.

Figure 3 presents a forest plot of the logit event estimates of contaminated meat items. Eight estimates were outside the confidence bounds of the regression line of the Galbraith plot (Figure 4), and the variation in prevalence estimates attributable to heterogeneity was substantially high (I^2^ = 76.6%). In a subgroup analysis by sample source, the I^2^ of the estimates in abattoirs and markets were 82.9% and 47.1% respectively. In a subgroup analysis by source species, the I^2^ was moderate to high in beef, goat meat and mutton from abattoirs and in minced beef from markets (Table 2). In a subgroup analysis of abattoir data, the I^2^ values were 85.9% for muscle and 78.9% for swab samples, but the pooled estimates (5.98% swab, vs. 5.05% muscle) did not differ significantly (*P* > 0.05). On the whole, differences in the meat handling practice, and the hygienic standards in slaughterhouses, and the transport means, the meat handling practice and storage facilities in the markets could have contributed to the heterogeneity of the study level estimates. The between studies variation in milk studies could have been due to differences in the occurrence of *Salmonella* among the study populations. In all instances, several factors including the breed, origin, and management of animals, and prior exposure of slaughtered animals to stress might have contributed to the within and between group variations.

**Table 2:** **Pooled prevalence of** ***Salmonella*** **in raw meat and milk**

**Source**	**Product**	**Pooled estimate**		**Heterogeneity**			
		**p (95% CI)**	**Z-** ***p***	**Q**	**Q-** ***p***	**Q/df**	**I** ^**2**^
Abattoir	Overall	5.57 (3.39, 9.01)	0.000	70.09	0.000	5.91	82.9
	Pork	3.57 (2.09, 6.06)	0.000	0.80	0.371	0.80	0.0
	Goat meat	3.86 (1.08, 23.20)	0.000	4.42	0.110	2.21	54.8
	Beef	4.53 (2.17, 9.25)	0.000	14.44	0.002	4.81	79.2
	Mutton	8.02 (3.04, 19.51)	0.000	7.98	0.019	3.99	74.9
	Camel^†^	21.02 (14.62, 29.26)	0.000				
Market	Overall	11.72 (9.71, 14.08)	0.000	18.91	0.041	1.89	47.1
	Minced beef	8.34 (4.75, 14.22)	0.000	11.85	0.008	3.95	74.7
	Mutton	11.86 (8.64, 16.07)	0.000	0.62	0.431	0.62	0.0
	Pork	12.59 (8.99, 17.35)	0.000	0.98	0.322	0.98	0.0
	Chicken	13.53 (10.93, 16.64)	0.000	0.65	0.721	0.33	0.0
Farm	Milk	10.76 (4.03, 25.71)	0.000	18.81	0.001	9.41	89.4

^†^The estimate was based on one study.

**Table 3:** **Number (%) of serotypes isolated from ruminant meat**

**Isolates**	**Serotypes**	**Total**	**Author**
Beef (n =91)^a^	*S.* Dublin	26 (28.57)	[25,26,37,38]
	*S.* Anatum	20 (21.98)	[25,26,37,38]
	*S.* Saintpaul	11(12.09)	[25,37,38]
	*S.* Newport	6 (6.59)	[27,28,37]
	*S.* Typhimurium	5 (5.50)	[26-28,37]
	*S.* Mishmarhaemek	5 (5.50)	[26]
Mutton/goat (n = 37)^b^	*S.* Infantis	17 (45.95)	[29,37,38]
	*S.* Newport	12 (32.43)	[37]
	*S.* Typhimurium	4 (10.81)	[30,37]
Camel (n = 25)^b^	*S.* Saintpaul	10 (40)	[34]
	*S.* Braenderup	8 (32)	[34]
	*S.* Muenchen	3 (12)	[34]

^a^Serotypes that accounted for 5% or more of the total isolates.

^b^Serotypes that accounted for 10% or more of the total isolates.

### Pooled estimates

Table 2 presents pooled estimates of *Salmonella* in animal products. The pooled prevalence estimates of contaminated pork, goat meat, beef and mutton carcasses in slaughter houses were 3.57%, 3.86%, 4.53% and 8.02%, respectively. The pooled estimates of contaminated minced beef, mutton, pork and chicken meat collected from markets were 8.34%, 11.86%, 12.59% and 13.53% respectively, and that of raw milk was 10.76%. All single study omitted estimates were within the 95% confidence bounds of the respective means. The source species significantly affected the occurrence of *Salmonella* in samples taken from slaughter houses (X^2^ = 8.57; df = 3; *P* < 0.05) but not in samples collected from markets (X^2^ = 7.11; df = 3; *P* > 0.05).

Although carcasses from apparently healthy animals are generally assumed to be free of *Salmonella,* contamination occurs in slaughter houses. The disparities in the extents of contamination could be due to differences in the skills of personnel in gut evisceration, carcass examination, carcass handling, and the hygienic standards of the slaughter houses, and there have been reports on the substandard knowledge, attitude and practice (KAP) of slaughterhouse personnel on food safety in Ethiopia [43,44]. Therefore, given the substandard KAP of personnel and the insanitary slaughterhouses’ environment in most cases in point, and a S*almonella* carrier prevalence of 7.07% in cattle to 43.81% in pigs [12], the likelihood of carcass contamination could be considerable. The differences in the prevalence of *Salmonella* by meat type could be ascribed to differences in the occurrence of the bacteria by source species that apparently influences the levels of contamination of slaughterhouses, personnel and slaughtering equipments. However, notwithstanding the effect of the source species, the lower prevalence of contaminated pork compared to other meat items could have been due to the hot water treatment of pig carcasses.

The odds of contaminated meat was more than twice higher in markets than in slaughterhouses [X^2^ = 43.54; *P* = 0.001; OR = 2.25 (95% CI = 1.75, 2.89)], and this could be due to further exposure of meat items to additional sources of contamination outside the slaughterhouses, and bacterial multiplication in faulty storages. The loading and unloading practice, the meat handling and processing practice, lack of adequate product holding facilities and power interruption could be implicated as potential factors that might have contributed to the higher prevalence of contaminated meat items in the markets. Moreover, in butcheries, carcasses are left in the open and exposed to environmental contaminants; the same utensils (cutting board and knives) are used for edible offal (tongue and rumen) and meats, and higher levels of contamination have been recorded in meat samples collected from retail markets, bars, restaurants and streets [45-47]. Furthermore, a higher bacterial count in retail outlets than in slaughterhouses [48], a positive association of bacterial count with market display temperature [49], and a linear association of temperature and cases of human salmonellosis [50] have been recorded elsewhere.

Beef, goat meat and milk are often consumed raw or undercooked and appear to be main vehicles of *Salmonella* to humans in Ethiopia. Consumption of raw mutton is less common, and pork is not popular due to either religious or cultural taboos or both. Chicken meat could be a potential source of contamination to other meat items with a greater risk during festive occasions where preparation of varieties of meat dishes (raw or undercooked and cooked) is a common practice in several households. In contrast, source attribution studies in Europe, USA, New Zealand and Japan attributed pigs for 10 to 23% [51], broilers for 48% [52], pigs for 60% [53] and layers for more than 50% [54] of the human illnesses, respectively.

### Serotypes

*S*. Dublin (28.57%), *S*. Infantis (45.95%) and *S*. Saintpaul (40%) were the most frequent serotypes isolated from beef, small ruminant and camel meat, respectively (Table 3). In pork and chicken meat, *S*. Newport (34.21%) and *S*. Braenderup (36%), respectively, were the most frequent isolates (Table 4). Although the preponderance of the serovars by meat type differ from reports elsewhere [55] and could vary across time [56], most have been isolated from samples taken from humans in Ethiopia: *S.* Braenderup, *S.* Newport*, S.* Dublin, *S*. Infantis, *S*. Saintpaul and *S.* Typhimurium from clinical samples [57,58], and *S.* Anatum*, S.* Newport and *S*. Dublin from personnel working in markets/abattoirs [25,37]. Therefore, despite limited data on isolates of human origin [14], and the absence of source attribution studies to human illnesses, the relative occurrence of the serovars implies their importance as potential causes of food-borne salmonellosis in Ethiopia.

**Table 4:** **Number (%) of serotypes isolated from non-ruminant meat**

**Isolates**	**Serotypes**	**Total**	**Author**
Chicken (n = 75)^a^	*S.* Braenderup	27 (36)	[35-37]
	*S.* Anatum	9 (12)	[35-37]
	*S.* Hadar	8 (10.67)	[36,37]
	*S.* Typhimurium	6 (8)	[35-37]
	*S.* Uganda	5 (6.67)	[35]
	*S.* Newport	4 (5.33)	[37]
	*S.* Saintpaul	4 (5.33)	[35]
Pork (n = 38)^b^	*S.* Newport	13 (34.21)	[33,37]
	*S.* Haifa	5 (13.16)	[37]

^a^Serotypes that accounted for 5% or more of the isolates.

^b^Serotypes that accounted for 10% or more of the isolates.

Of the market isolates, resistance to three or more antimicrobials (multi-drug resistance, MDR) was recorded in *S.* Braenderup [35,37,59,60], *S.* Newport, *S*. Haifa [37], *S*. Anatum [35,57], *S*. Saintpaul, *S*. Roughform, *S*. Uganda [59] and *S*. Typhimurium [59-61]. Moreover, more than half of the milk isolates (25/42), [40-42], and 19.12% of the isolates of slaughtered ruminant origin were MDR, and certain strains were reportedly resistant to drugs uncommonly used in the veterinary sector [62]. Furthermore, *Salmonella* has been isolated from personnel in contact with animals or animal products [14], and the proportion of MDR isolates of human origin was estimated at 79.56% [63]. In general, regardless of the limited data on the drug resistance profiles of *Salmonellae* isolated from animal products, the risk of acquiring MDR *Salmonella* through the consumption of raw or undercooked animal products appears considerable.

### Implications and limitations

The pooled estimates demonstrate the level of contaminated animal products meant for public consumption and imply the need for strict observations of food safety in slaughterhouses and markets. Policy makers could make use of the estimates as inputs to enforce food safety measures and reduce the risks of contamination of animal products. The limited number of studies was the main constraint to calculate robust pooled estimates by sample source and type. Therefore, as most studies have been carried out in slaughterhouses and markets in Addis Ababa and the surrounding towns, the pooled prevalence estimates of contaminated meat items are more appropriate to bigger urban than to rural and smaller settings of the country.

## Conclusions

The estimates demonstrate the extent of contamination, and entail the need for quality assurance programs to ensure the safety of animal products to consumers. Food safety educational programs in slaughterhouses and markets, and public education as regards the risks of consumption of raw or undercooked animal products are important lines of defense against *Salmonella* and other food-borne pathogens.

## Additional file

Additional file 1:
**PRISMA checklist.**
